# Synthesis of an amphiphilic vancomycin aglycone derivative inspired by polymyxins: overcoming glycopeptide resistance in Gram-positive and Gram-negative bacteria in synergy with teicoplanin in vitro

**DOI:** 10.1038/s41598-022-24807-0

**Published:** 2022-12-03

**Authors:** Zsolt Szűcs, Ilona Bereczki, Ferenc Fenyvesi, Pál Herczegh, Eszter Ostorházi, Anikó Borbás

**Affiliations:** 1 Department of Pharmaceutical Chemistry, University of Debrecen, Egyetem Tér 1, 4032 Debrecen, Hungary; 2 Department of Pharmaceutical Technology, Faculty of Pharmacy, University of Debrecen, Nagyerdei Körút 98, 4032 Debrecen, Hungary; 3 Department of Medical Microbiology, Semmelweis University, Nagyvárad Tér 4, 1089 Budapest, Hungary

**Keywords:** Drug discovery and development, Synthetic chemistry methodology

## Abstract

Gram-negative bacteria possess intrinsic resistance to glycopeptide antibiotics so these important antibacterial medications are only suitable for the treatment of Gram-positive bacterial infections. At the same time, polymyxins are peptide antibiotics, structurally related to glycopeptides, with remarkable activity against Gram-negative bacteria. With the aim of breaking the intrinsic resistance of Gram-negative bacteria against glycopeptides, a polycationic vancomycin aglycone derivative carrying an *n*-decanoyl side chain and five aminoethyl groups, which resembles the structure of polymyxins, was prepared. Although the compound by itself was not active against the Gram-negative bacteria tested, it synergized with teicoplanin against *Escherichia coli, Pseudomonas aeruginosa* and *Acinetobacter baumannii*, and it was able to potentiate vancomycin against these Gram-negative strains. Moreover, it proved to be active against vancomycin- and teicoplanin-resistant Gram-positive bacteria.

## Introduction

Glycopeptide antibiotics are used for treating several types of infections caused by Gram-positive bacteria, such as methicillin resistant *Staphylococcus aureus* (MRSA), vancomycin resistant enterococci (VRE) etc. either in monotherapy or in combination with other antibiotics^1,2^. Vancomycin is also used *per os* in *C. difficile* infections^3,4^. Semisynthetic glycopeptides such as telavancin or oritavancin are indicated for the treatment of acute bacterial skin and skin structure infections (ABSSSI)^5,6^. The fundamental mechanism of action of these compounds is the inhibition of bacterial cell-wall synthesis by binding to the d-Ala-d-Ala termini of peptidoglycan precursors with hydrogen bonds^7,8^.

According to several healthcare sources (e.g. CDC, Centers for Disease Control and Prevention)^9^ and clinical experience, Gram-negatives are at the top of the list of the most dangerous bacteria today due to a very broad spectrum of resistance mechanisms, which sometimes makes it impossible to treat patients effectively^10,11^. These pathogens are intrinsically resistant to glycopeptide antibiotics because of the presence of an outer membrane, which consists of phospholipids, negatively charged lipopolysaccharides (LPS), porins and Braun's lipoprotein. The relatively large glycopeptides are unable to cross the outer membrane either by diffusion or by transport through the porins to reach the peptidoglycan.

Our goal was to prepare a semisynthetic glycopeptide which can overcome this intrinsic resistance. Some research groups have already synthesized such derivatives^12–14^ (e.g. several amide derivatives of teicoplanin and its aglycone, where different polyamines were coupled to the *C*-terminal carboxyl group)^15^. The aglycone derivatives were notably more effective, possibly because of their smaller molecular size. Other research groups prepared *C*-terminal amide derivatives of vancomycin using alkyl amines with quaternary ammonium groups^16,17^, a single amino acid arginine^18,19^ or LPS-binding peptides^20^ to vancomycin and these manipulations have successfully endowed the compounds with activity against Gram-negative bacteria.

Our idea was considerably different than those above. The synthetic goal was a compound that resembles an antibiotic which is already proven to be effective against Gram-negative bacteria.

**Figure 1:**
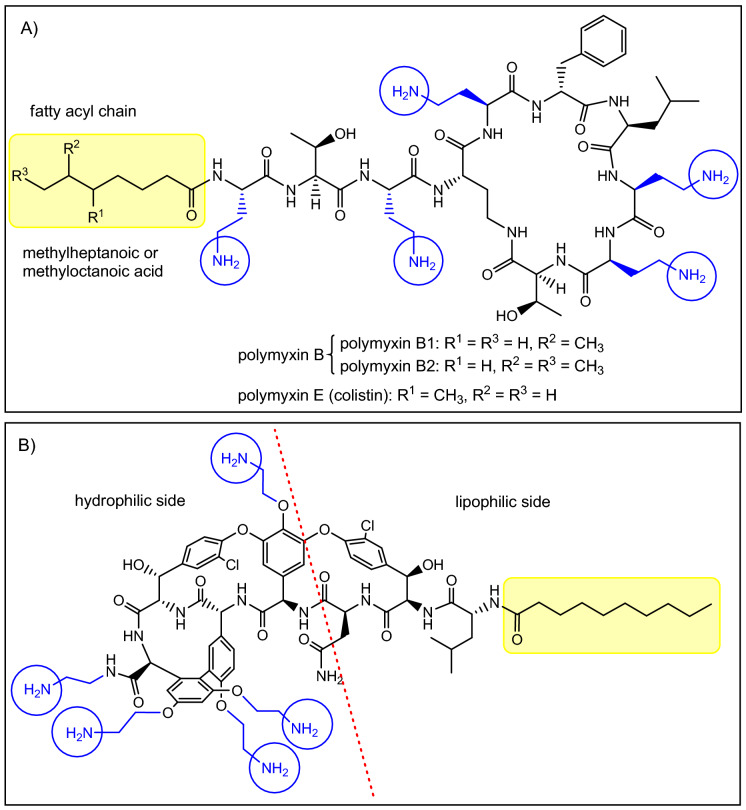
(**A**) The structure of polymyxins. (**B**) Target compound with five primary amino groups and a fatty acyl side chain.

Among currently used antibiotics, polymyxins^21,22^ (Fig. 1A) are the most relevant structurally related compounds to glycopeptides as—despite significant differences—both are peptides in the 1000–2000 Dalton MW range. These polycationic antibiotics are remarkably effective against Gram-negative bacteria, however they are also highly toxic, which is the reason why they are used only as a last resort^22^. The primary mechanism of action of polymyxins is related to the bacterial outer membrane. We anticipated that this cellular structure should be an ideal target for a polycationic glycopeptide derivative as well. As a matter of fact, targeting intracellular structures with glycopeptides would be less reasonable, as despite any synthetic modifications, the derivatives would be most likely still too large to cross both the outer and inner bacterial membranes. Furthermore, these antibiotics are inherent cell-wall synthesis inhibitors having no significant action on intracellular targets.

Polymyxins have two key structural components that are essential to their activity^23^. The five aliphatic amino groups in protonated form allow the molecules to bind to the LPS of the Gram-negative outer membrane, displacing the stabilizing Ca^2+^- and Mg^2+^-ions, while the simultaneous presence of a fatty acyl side chain provides an amphiphilic character, which leads to the disruption of the membrane. Glycopeptides possess a substantially dissimilar structure with much less flexibility and different spatial organization of amino acids, which seem to be key in the antibacterial activity of polymyxins. However, even lipophilic cholic acid derivatives with multiple primary amino/guanidine groups were reported to permeabilize the membrane^24^. This effect may be comparable to polymyxin B nonapeptide (polymyxin B missing the fatty acyl side chain), depending on the structure of said compounds. Polymyxin B nonapeptide, despite not being active, has been shown to help otherwise ineffective antibiotics to cross the membrane and act accordingly, thanks to its cationic structure^12^. Considering these, it is probably not surprising, that glycopeptides mentioned above with a positively charged polyamine chain or quaternary ammonium group showed activity against Gram-negative strains^12,16,17^.

We hypothesized that the incorporation of amino groups on multiple sites and an acyl chain into a glycopeptide antibiotic might broaden its activity spectrum toward Gram-negative strains. In addition, based on the literature^25,26^ and our own results^27,28^, we assumed that such compound, which can be considered a polymyxin analog on the one hand, and a cationic glycopeptide derivative on the other hand, can also be effective against Gram-positive bacteria. We have shown that the introduction of cationic guanidine groups endows the glycopeptide antibiotic teicoplanin with strong activity against resistant Gram-positive strains^27,28^. In addition, there is increasing evidence that polymyxins and related compounds are suitable for the treatment of Gram-positive bacterial infections, presumably because negatively charged teichoic acids produced by Gram-positive bacteria can be targeted by polymyxins^23,25,26^.

## Results and discussion

For the synthetic modification, the glycopeptide of choice was vancomycin, as it is a relatively inexpensive and easily accessible starting material. The target molecule we planned to prepare (Fig. 1B) has the structural elements that could hypothetically provide antibacterial activity against Gram-negative bacteria: (a) multiple amino groups to interact with the LPS as seen in the case of polymyxins or polyamines, (b) a lipophilic side chain, separated from amino groups in space, to make the compound sufficiently amphiphilic to permeabilize the membrane. In theory, this could result in an antibiotic with a “2-in-1” action, interacting with the outer membrane in the first step, making it permeable. Then by self-promoted uptake (like polymyxins) it could further disturb the membrane, and most importantly, reach the cell-wall and exert its inherent cell-wall synthesis inhibitor effect.

**Figure 2:**
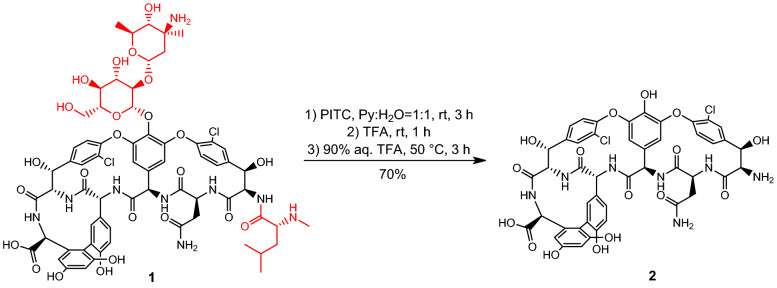
Synthesis of vancomycin aglycone hexapeptide (VAHP) **2**.

**Figure 3:**
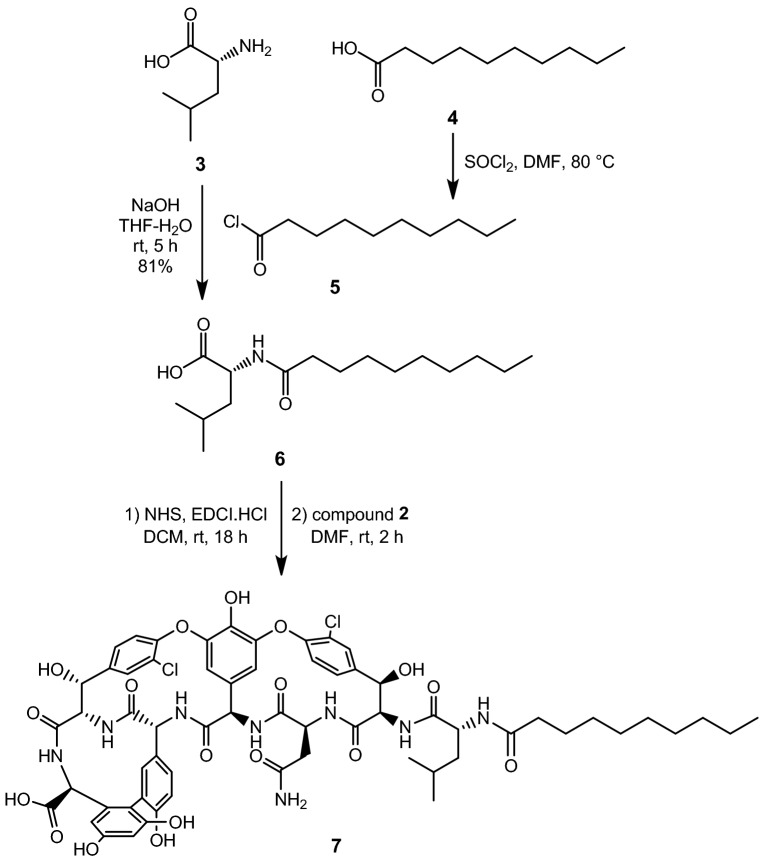
Acylation of VAHP.

The first two relevant modifications were the removal of the disaccharide moiety to decrease the molecular size and the attachment of the fatty acyl side chain. These were carried out in a four-step procedure (Figs. 2 and 3).

First, vancomycin (**1**) was submitted to the two-step Edman degradation to remove the *N*-Me-d-Leu moiety, as direct acylation of the *N*-terminus proved to be ineffective. After the reaction of **1** with phenylisothiocyanate (PITC), the crude phenylthiocarbamoyl derivative was treated with TFA to remove the *N*-terminal amino acid, then the disaccharide was hydrolyzed in the same flask at elevated temperature to yield vancomycin aglycone hexapeptide (VAHP) **2**^29,30^. After workup the crude product was suitable for further use (Fig. 2).

Next, d-leucine **3** was *N*-acylated with decanoyl chloride **5**, prepared from decanoic acid **4**, to yield *N*-decanoyl-d-leucine **6**. From this material, its *N*-hydroxysuccinimide (NHS) active ester was prepared using a standard procedure with 1-ethyl-3-(3-dimethyllaminopropyl)carbodiimide hydrochloride (EDCI.HCl) and *N*-hydroxysuccinimide. The active ester was suitable for acylation without any purification. The reaction of crude VAHP **2** and the NHS ester of **6** yielded the acylated derivative **7**, which was used in the next step after simple workup (Fig. 3).

**Figure 4:**
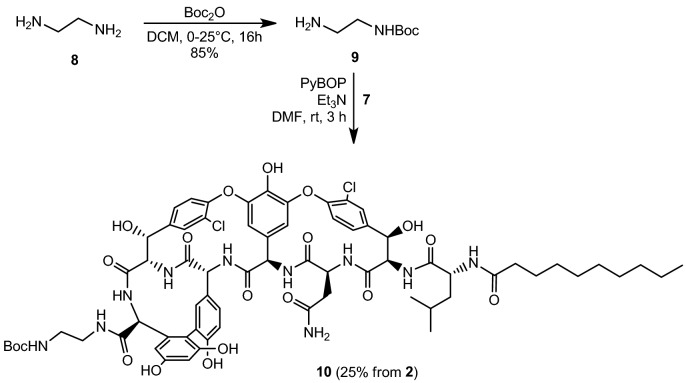
Amide formation at the *C*-terminal of vancomycin aglycone derivative **7**.

The following task was to link the amine-containing units to the peptide. As the polymyxins have five amino groups, we decided that the vancomycin derivative should carry five primary amino groups, as well. First, *N*-Boc-ethylenediamine **9** was prepared using an excess of ethylenediamine **8** and Boc_2_O^31^. After workup, PyBOP mediated amide formation was carried out on **7** using **9** as the amine component (Fig. 4). At this point, the amide product **10** was purified by column chromatography.

**Figure 5:**
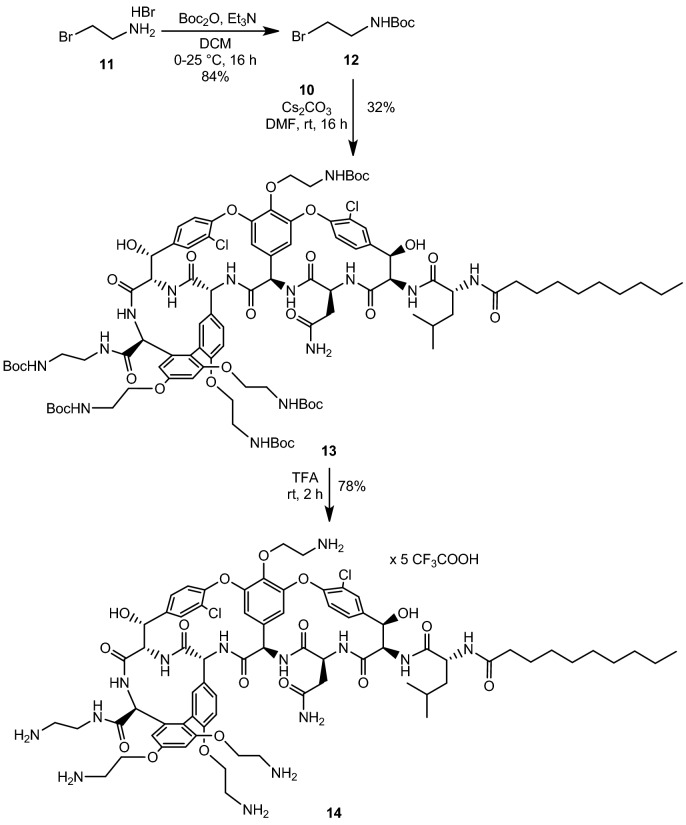
The alkylation of phenolic OH-groups and global deprotection.

Next, *N*-Boc-bromoethylamine **12** was prepared from bromoethylamine hydrobromide **11** using Boc_2_O^32^. The alkylation^33^ of **10** in the presence of a large excess of **12** and Cs_2_CO_3_ gave the tetraalkylated product **13** (Fig. 5). After column chromatography, further purification was accomplished by preparative TLC to remove residual impurities from the previous reactions. In the final step, the protecting groups were removed by TFA, and the product **14** was isolated as the trifluoroacetate salt after reversed phase column chromatography.

**Figure 6:**
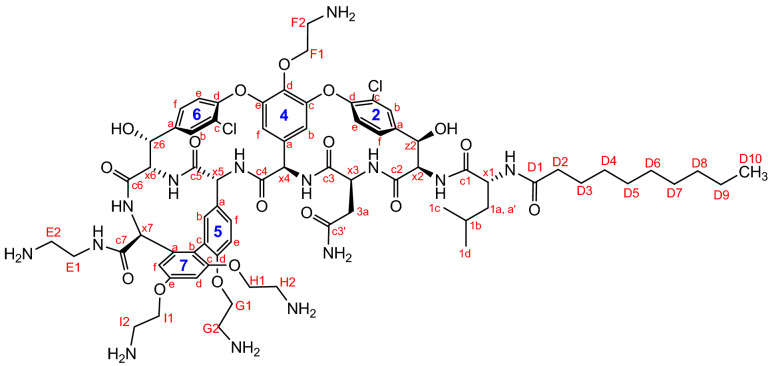
Atom numberings for compound **14** for NMR assignment.

The structure of compound **14** was confirmed by different NMR techniques, including COSY, HSQC, HMBC and NOESY experiments. (The NMR data are collected in Tables 1 and 2, atom numbering is shown in Fig. 6). All spectra are available in the supplementary information S1.

**Table 1:** NMR assignments for the vancomycin aglycone part of compound **14.**

Assignment	^1^H	^13^C	Assignment	^1^H	^13^C
**Vancomycin aglycone**					
c1	–	175.16	6c	–	126.69
c3	–	173.4	2f	7.72	125.14
c7	–	172.9	2e	7.48	124.59
c4	–	170.3	6e	7.34	124.02
c5	–	168.8	5c	–	123.23
c6	–	162.9	7b	–	121.52
c2	–	162.6	5e	7.24	115.31
7e	–	158.8	7f	6.62	107.97
7c	–	156.8	4b	5.60	105.82
5d	–	155.5	4f	5.54	104.65
(4e)	–	152.70	7d	6.92	101.43
(4c)	–	151.55	z6	5.63	71.4
2d	–	150.22	z2	5.55	70.07
6d	–	149.55	x6	4.29	63.22
6a	–	140.19	x2	4.86	59.10
2a	–	137.8	x7	4.69	58.56
7a	–	135.94	x4	5.85	55.02
5b	–	134.86	x5	4.54	54.53
4d	–	134.29	x1	4.19	53.88
4a	–	133.4	x3	4.85	51.69
2c	–	129.25	1a, aʹ	1.81, 1.66	38.37
2b	7.48	129.21	3a	2.61	37.82
5a	–	128.06	1b	1.74	24.32
6b	7.68	127.96	(1c)	0.99	22.06
5f	7.32	127.73	(1d)	0.91	20.14
6f	7.60	127.18

**Table 2:** NMR assignments for the side chains of compound **14.**

*n*-Decanoyl side chain			2-Aminoethyl side chains		
Assignment	^1^H	^13^C	Assignment	^1^H	^13^C
D1	–	178.8	E1	3.81, 3.65	37.2
D2	2.17	35.44	E2	3.26	38.78
D3	1.43	25.2	F1	4.68	69.96
D4	1.19–1.29	28.06	F2	3.56	39.51
D5		28.29	G1	4.32	65.12
D6		28.38	G2	3.35	38.62
D7		28.68	H1	4.23, 4.29	65.5
D8	1.23	31.05	H2	3.22	38.53
D9	1.26	21.91	I1	4.38	64.23
D10	0.86	13.28	I2	3.52	38.80

## Biological evaluation

Compound **14** was tested on eight Gram-positive and four Gram-negative strains using teicoplanin and vancomycin as reference antibiotics (Table 3).

**Table 3:** Antibacterial activity.

Bacterial strains		Antibiotic		
		Teicoplanin	Vancomycin (**1**)	**14**
**MIC in μg/mL**				
Gram-positive	*Bacillus subtilis* ATCC 6633	0.5	0.5	4
	*Staphylococcus aureus* MSSA ATCC 29213	0.5	0.5	8
	*Staphylococcus aureus* MRSA ATCC 33591	0.5	0.5	8
	*Staphylococcus epidermidis* ATCC 35984 biofilm	4	2	4
	*Staphylococcus epidermidis* mecA	16	4	2
	*Enterococcus faecalis* ATCC 29212	1	1	16
	*Enterococcus faecalis* 15376 VanA	256	256	8
	*Enterococcus faecalis* ATCC 51299 VanB	0.5	128	32
Gram-negative	*Klebsiella pneumoniae* ST258 clone K 160/09	256	256	256
	*Pseudomonas aeruginosa* ATCC 27853	256	256	128
	*Acinetobacter baumannii* ATCC BAA1605	128	128	128
	*Escherichia coli* ATCC 25218	256	256	128

*ATCC* American type culture collection, *MSSA* methicillin-sensitive *Staphylococcus aureus*, *MRSA* methicillin-resistant *Staphylococcus aureus*, *mecA* mecA gene expression in *Staphylococcus*, *VanA* vanA gene positive, *VanB* vanB gene positive, *MIC* minimum inhibitory concentration.

The new polymyxin-like compound showed a surprising profile of activity against Gram-positive strains, with a remarkable effect against resistant strains and only a moderate effect against non-resistant strains. The VanB type (vancomycin-resistant, teicoplanin-sensitive) *E. faecalis* was more susceptible to compound **14** than to vancomycin (32 vs. 128 μg/mL). More curiously, the vancomycin and teicoplanin resistant VanA type *E. faecalis* was even more susceptible with a MIC of 8 μg/mL. VanA phenotype resistance is the most frequently encountered glycopeptide antibiotic resistance in enterococci, which causes cell wall reprogramming, resulting in incorporation of d-alanine-d-lactate or d-alanine-d-serine into the peptidoglycan precursors instead of the natural d-alanine-d-alanine (d-Ala-d-Ala)^8^. This modification in the dipeptide unit greatly reduces the affinity of glycopeptide antibiotics teicoplanin and vancomycin to peptidoglycan. We assume that, on the one hand, compound **14** can bind strongly to the cell membrane through its lipophilic decanoyl side chain, and on the other hand, due to synthetic modifications of the peptide core, its binding affinity towards the modified peptidoglycan precursors might be higher than that of the native glycopeptide antibiotics. Together, these effects may result in the high activity of compound **14** against vanA *E. faecalis*.

The *S. epidermidis* strain carrying the mecA gene, which is also teicoplanin resistant, was also more susceptible to the new derivative than to vancomycin or teicoplanin.

At the same time, bacteria that are not glycopeptide resistant, such as MSSA, MRSA and *E. faecalis* (ATCC 29212) were found to be generally less (or equally) susceptible to compound **14** than to the reference antibiotics. The most striking example is probably the comparison of MIC values obtained for vancomycin sensitive *E. faecalis* ATCC 29212 and vancomycin/teicoplanin resistant VanA *E. faecalis.* Compound **14** is 16 times less active against the vancomycin sensitive strain, than vancomycin, but it is 32 times more potent against the resistant VanA strain. We assume that the reduced activity against vancomycin-susceptible strains can be explained by the highly modified aglycon part, as a result of which the binding affinity/accessibility of **14** to the d-Ala-d-Ala peptidoglycan precursors decreases.

Unfortunately, compound **14** did not exert activity against any of the Gram-negative strains tested. Despite this inactivity, we hypothesized that due to its polycationic structure it should have membrane activity, so it may potentiate glycopeptide antibiotics against Gram negative bacteria. It is worth noting here that the synergistic activity of glycopeptide antibiotics vancomycin, teicoplanin and telavancin in combination with colistin (polymyxin E) against Gram-negative bacteria has been previously reported^12,34–37^. However, only *Acinetobacter baumanniii* was included in the synergistic studies with teicopanin^34^ and vancomycin^35^, and there are no results in the literature on the activity of these combinations against other Gram-negative strains.

To determine the activity profile of teicoplanin and vancomycin in combination with compound **14**, combined susceptibility tests were perfomed against five Gram negative strains including *Escherichia coli, Pseudomonas aeruginosa, Acinetobacter baumannii* and *Klebsiella pneumoniae* bacteria. The combined effects of compound **14** and the glycopeptide antibiotics were determined by conventional checkerboard assays (images of the chequerboard assay for synergistic combinations are shown in Supplementary Information S1).

Upon combining compound **14** with teicoplanin against *Escherichia coli, Pseudomonas aeruginosa* and *Acinetobacter baumannii* marked synergy was observed with ΣFIC = 0.2578, 0.375 and 0.2656, respectively. When vancomycin was combined with **14**, additive interactions were detected against these strains (ΣFIC = 0.5078, 0.5625 and 0.75, respectively). Combining **14** with teicoplanin or vancomycin against *Klebsiella pneumoniae* resulted in neither synergy nor additive activity (Table 4).

**Table 4:** Adjuvant potency of compound **14** in combination with teicoplanin and vancomycin.

Bacterial strains	Antibiotic (combined with **14**)	MIC of antibiotic alone (μg/mL)	MIC of antibiotic in comb. (μg/mL)	MIC of **14** alone (μg/mL)	MIC of **14** in comb. (μg/mL)	FIC index	Combined effect
*Escherichia coli* ATCC 25218	Teicoplanin	256	2	256	32	0.2578	Synergy
*Escherichia coli* ATCC 25218	Vancomycin		2		64	0.5078	Additive
*Pseudomonas aeruginosa* ATCC 27853	Teicoplanin	256	32	256	32	0.375	Synergy
*Pseudomonas aeruginosa* ATCC 27853	Vancomycin		16		64	0.5625	Additive
*Acinetobacter baumannii* ATCC BAA1605	Teicoplanin	128	32	128	2	0.2656	Synergy
*Acinetobacter baumannii* ATCC BAA1605	Vancomycin		64		32	0.75	Additive
*Klebsiella pneumoniae* ST258 clone K 160/09	Teicoplanin	256	256	256	256		None
*Klebsiella pneumoniae* ST258 clone K 160/09	Vancomycin		256		256		None

**Figure 7:**
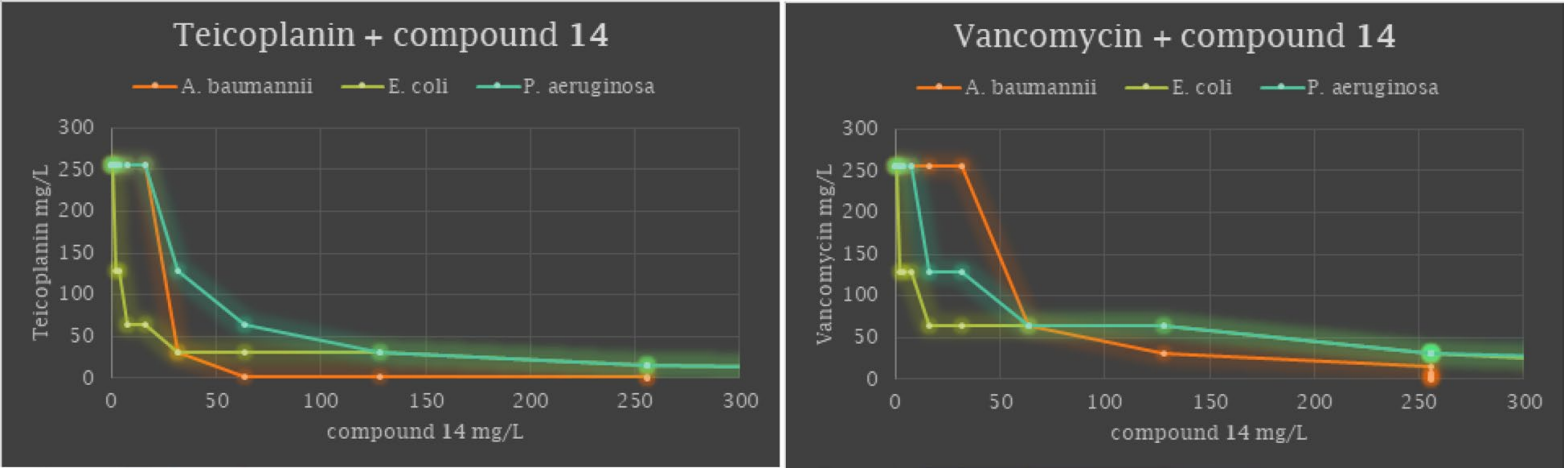
Isobolograms for combinations of compound **14** with teicoplanin and vancomycin tested at various ratios against *Acinetobacter baumannii* ATCC BAA1605, *Escherichia coli* ATCC 25218 and *Pseudomonas aeruginosa* ATCC 27853.

The additive or synergistic effects of the compound mixtures are visualized by isobologram graphs of the combinations, generated from the FIC values (Fig. 7).

**Figure 8:**
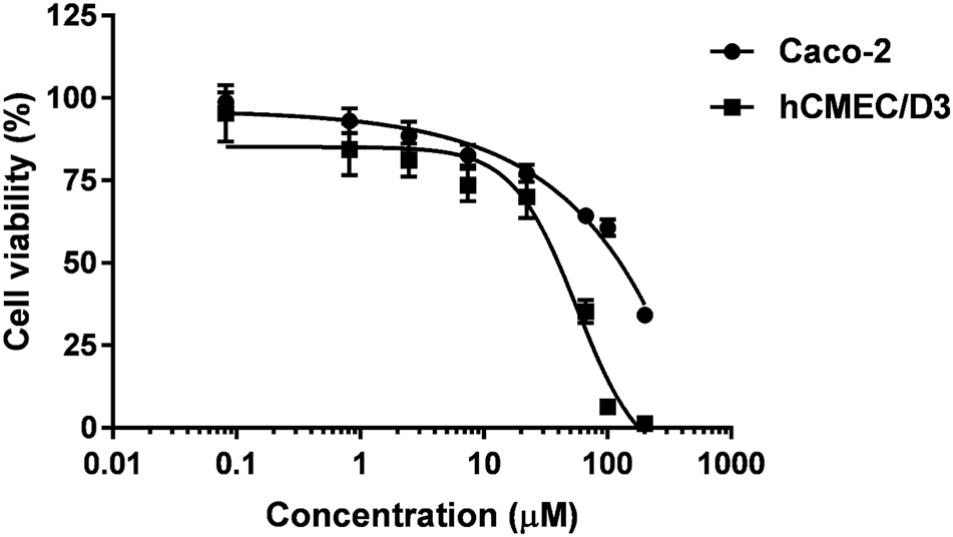
Dose-dependent cytotoxicity of compound **14** towards Caco-2 and hCMEC/D3 cells.

The cationic modification performed on vancomycin could significantly alter the toxicity profile of the modified derivative. The potential toxicity of compound **14**, was tested against Caco-2 and hCMEC/D3 cell lines. Caco-2 cells are human intestinal epithelial cells, while hCMEC/D3 cell line is a human brain capillary endothelial cell line. The two cell types were chosen to test the biocompatibility of antibiotics on different barriers in the body after the administration through the two major application routes, the oral or intravenous application. The cationic vancomycin derivative **14** showed a dose-dependent cytotoxicity both on Caco-2 and hCMEC/D3 cells, but fortunately low toxicity was observed in both cases. The IC_50_ value was 56.22 ± 7.74 µM (116.29 ± 16 µg/mL) for hCMEC/D3 cells, and > 100 µM (> 206.86 µg/mL) for Caco-2 cells (Fig. 8).

**Figure 9:**
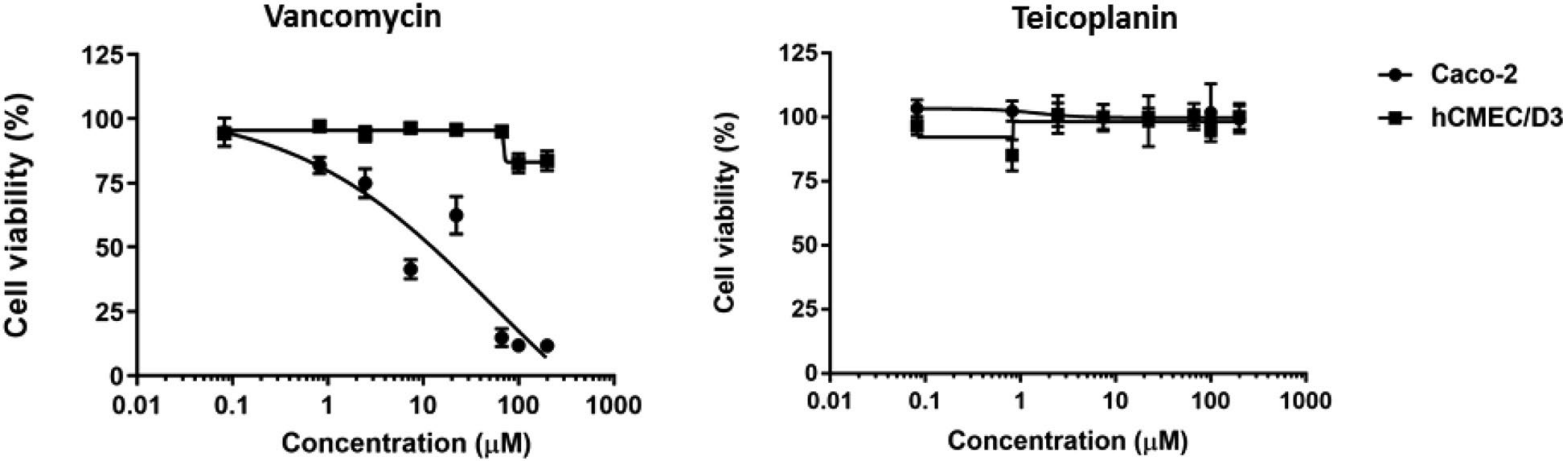
Cytotoxicity of glycopeptide antibiotics vancomycin and teicoplanin towards Caco-2 and hCMEC/D3 cells.

For comparison, the effect of vancomycin and teicoplanin on the viability of the two cell types was also tested (Fig. 9). Teicoplanin did not show cytotoxic effect on Caco-2 or hCMEC/D3 cell lines between 0.082 and 200 µM (0.155–375.94 µg/mL). Interestingly Caco-2 cells were more sensitive to vancomycin than hCMEC/D3 cells. The IC_50_ value of vancomycin on Caco-2 cells was 54.24 µM (78.65 µg/mL).

**Figure 10:**
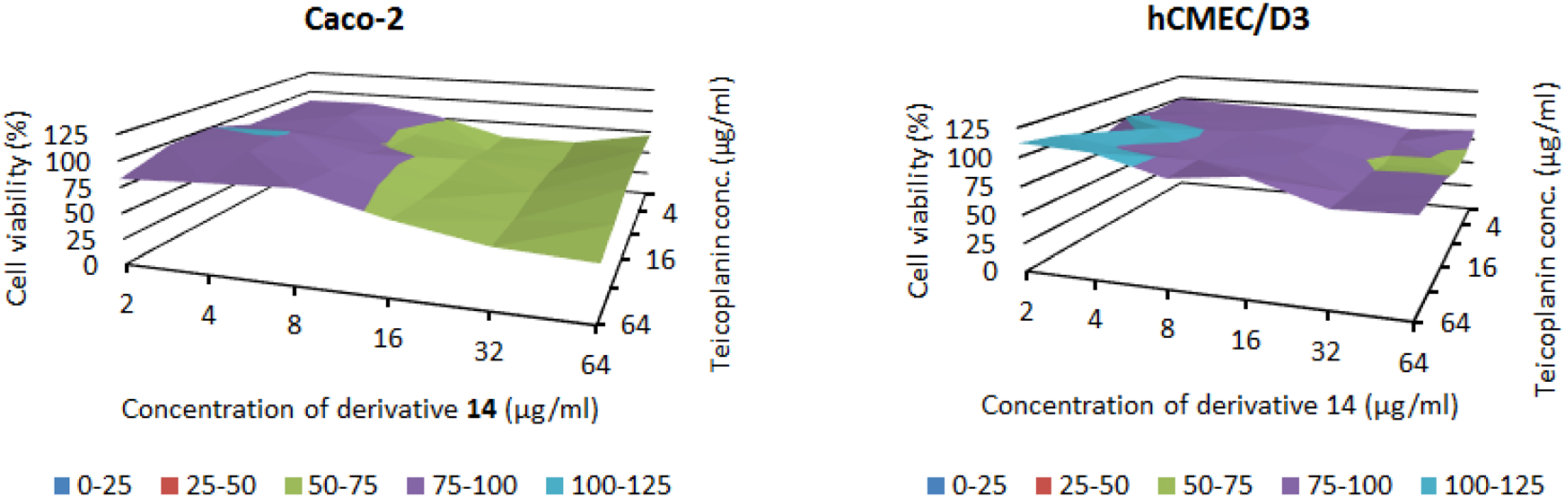
Cytotoxic effect of teicoplanin in combination with compound **14** towards Caco-2 and hCMEC/D3 cells, measured by checkerboard method.

**Figure 11:**
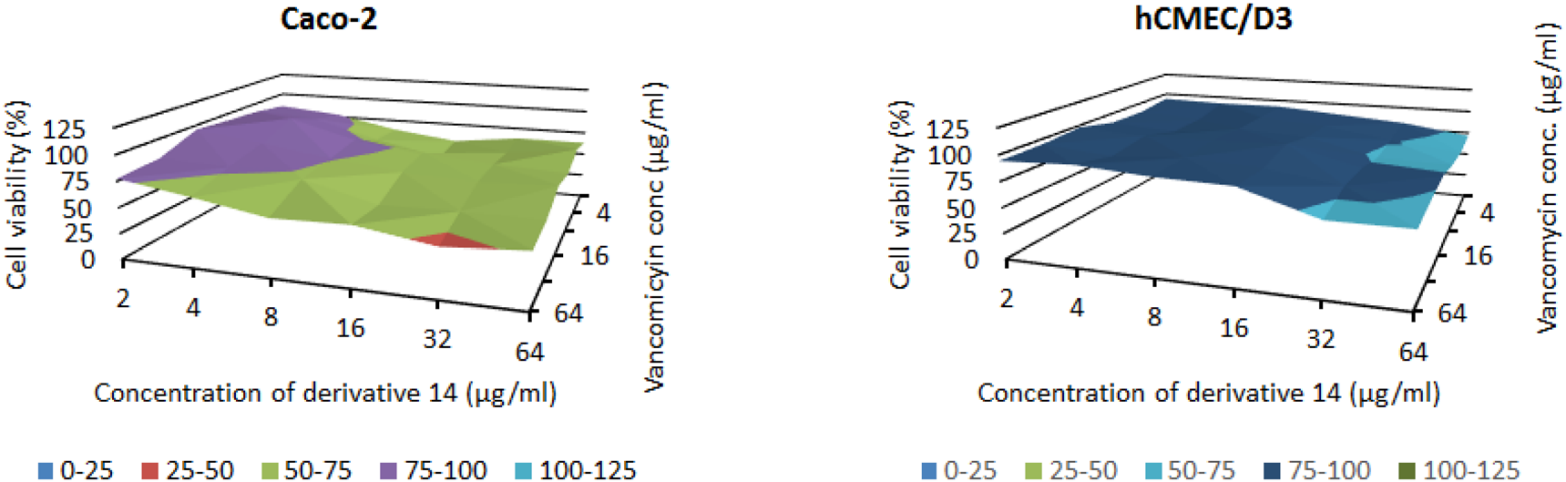
Cytotoxic effect of vancomycin in combination with compound **14** towards Caco-2 and hCMEC/D3 cells, measured by checkerboard method.

Finally, to ascertain the biocompatibility of the compound mixtures, cytotoxicity assays were performed with the combinations in checkerboard layout. The combinations of derivative **14** with teicoplanin (Fig. 10), and with vancomycin (Fig. 11) showed cytotoxicity in a lower concentration range, compared to the effect of antibiotics alone, however, they inhibited only partially the cell viability between 2 and 64 µg/mL. Importantly, there are combinations, which did not show or only minor toxicity on the cells, but had synergistic effects on bacteria. The combination teicoplanin : compound **14**—32:2 µg/mL was not toxic on either cell line, and the combination teicoplanin : compound **14**—2:32 µg/mL only slightly decreased the viability of hCMEC/D3 cells to 81.4%. These combinations had synergistic effects on *Acinetobacter baumannii* and *Escherichia coli*, respectively. However, it is important to note, that the possibility of clinical use of the combination against *A baumannii* is questionable, since concentration of teicoplanin effective in synergism would probably result in toxic side effects in vivo.

## Conclusions

The new polymyxin-like vancomycin aglycone derivative **14** showed remarkable activity against resistant Gram-positive bacteria but exerted only moderate activity against non-resistant strains. The reason for this seemingly controversial activities against Gram-positive strains might be a considerably different mode of action of the new derivative compared to teicoplanin or vancomycin. The diminished activity against the vancomycin sensitive strains could be due to the heavily modified aglycone part. Plausible explanations could be e.g. the binding pocket becoming less accessible for the peptidoglycan precursors due to steric hindrance and/or the slightly altered orientation of the binding pocket due to intramolecular interactions. Concerning the enhanced activity against vancomycin and teicoplanin resistant bacteria, we assume that the sufficiently strong binding affinity of compound **14** to both modified peptidoglycan precursors and the cell membrane may play a role in the mechanism of action. At the same time, a polymyxin-like effect, based on interaction with the cell wall teichoic acids of Gram-positive bacteria, cannot be ruled out either^25,26^.

Despite a thoughtful and reasonable design, compound **14** alone did not show antibacterial activity against the tested Gram-negative strains. Uhl and co-workers recently reported similar results when modifying vancomycin with a polycationic peptide; the modification was found to be effective against resistant Gram-positive bacteria but did not lead to activity against Gram-negative strains^38^.

The main finding of our study is that compound **14** was able to synergize with teicoplanin and strongly potentiated vancomycin against Gram-negative bacteria including *Escherichia coli, Pseudomonas aeruginosa* and *Acinetobacter baumannii*, demonstrating the ability of compound **14** to compromise the integrity of the outer membrane of Gram-negative strains.

Although earlier reports of combination of colistin with teicoplanin and vancomycin exist, these studies were limited to determination of the activity against *A. baumanii*^34,35^. Herein, we performed an extended combined susceptibility study by incorporating five Gram negative strains.

We observed relevant differences in potentiation efficacy against different bacterial strains with the same antibiotic. Although this phenomenon seems surprising, similar results have been reported by the Wareham group in the study of the synergistic effect of telavancin and colistin^36^. The membrane perturbation ability of compound **14**, which plays a key role in the potentiating effect, is associated with its binding to bacterial lipopolysaccharides. The composition of the lipopolysaccharide is different in the bacteria involved the present study, and the phosphorylation pattern of the lipopolysaccharides may also be different. We hypothesize that compound **14** interacts with different affinities with different lipopolysaccharides, leading to different membrane perturbation ability and thus different antibacterial activity.

Our results provides further evidence that using glycopeptide antibiotics in combination with membrane-active agents is a viable and resourceful strategy to repurpose them against Gram-negative bacteria.

## Experimental

### General information

Vancomycin hydrochloride was a gift from TEVA Pharmaceutical Industries Ltd. (Debrecen, Hungary). TLC was performed on Kieselgel 60 F_254_ (Merck) with detection either by immersing into ammonium molybdate-sulfuric acid solution followed by heating or by using Pauly’s reagent for detection. Flash column chromatography was performed using Silica gel 60 (Merck 0.040–0.063 mm) and Silica gel 60 silanized (0.063–0.200 mm). The ^1^H NMR (360, 400 and 500 MHz), ^13^C NMR (90, 100 and 125 MHz) and 2D NMR spectra were recorded with a Bruker DRX-360, a Bruker DRX-400 and a Bruker Avance II 500 spectrometers at 300 K. Chemical shifts are referenced to Me_4_Si and to the solvent residual signals. MALDI-TOF MS measurements were carried out with a BIFLEX III mass spectrometer (Bruker, Bremen, Germany) or a Bruker Autoflex Speed mass spectrometer equipped with time-of-flight (TOF) mass analyzer. 2,5-Dihydroxybenzoic acid (DHB) was used as matrix and CF_3_COONa as cationizing agent in DMF. For analytical RP-HPLC a Waters 2695 Separations Module (Waters Corp., Milford, USA) was used. The separations were carried out on a VDSphere PUR 100 C18-M-SE, 5 μm, 150 × 4.6 mm column at an injection volume of 10 μL, using a flow rate of 1.0 mL/min with a Waters 2996 DAD set at 254 nm and a Bruker MicroTOF-Q type Qq-TOF MS instrument (Bruker Daltonik, Bremen, Germany) as detectors. The following system was used for the elution: Solvent A: water:MeCN 9:1 + 0.0025%v/v TFA and Solvent B: MeCN. Gradient elution: from 0% of B to 20% from 0 to 10 min then from 20% of B to 80% from 10 to 30 min and 80% of B from 30 to 35 min. The MicroTOF-Q mass spectrometer was equipped with an electrospray ion source. The mass spectrometer was operated in positive ion mode with a capillary voltage of 3.5 kV, an endplate offset of − 500 V, nebulizer pressure of 1.8 bar, and N_2_ as drying gas with a flow rate of 9.0 l/min at 200 °C. The mass spectra were recorded by means of a digitizer at a sampling rate of 2 GHz. The mass spectra were calibrated externally using the exact masses of clusters [(NaTFA)_n_+TFA]^+^ from the solution of sodium trifluoroacetate (NaTFA). The spectra were evaluated with the DataAnalysis 3.4 software from Bruker.

The routine antibacterial evaluations were carried out as it was described in our previous publication^39^. Antimicrobial synergy was determined by evaluating the fractional inhibitory concentration (FIC) index and was characterized by a conventional checkerboard assay^40^. Bacteria grown to mid-logarithmic phase in MHB were pre-incubated with serially diluted concentrations of teicoplanin or vancomycin and compound **14**. The sum of the FICs (ΣFIC) was calculated with the equation ΣFIC = FICA + FICB = (CA/MICA) + (CB/MICB), where MICA and MICB are the MICs of antimicrobial A and B alone, respectively, and CA and CB are the inhibitory concentrations of the drugs when combined, respectively. Synergy was defined as a ΣFICs ≤ 0.5 and additive activity was defined as a ΣFICs > 0.5 ≤ 1.0.

The human immortalized hCMEC/D3 cells (Merck KGaA, Darmstadt, Germany, Cat. # SCC066) were cultured in EndoGRO-MV Complete Culture Media (supplemented with the components of the kit and fibroblast growth factor 2 (FGF-2) at 1 ng/mL final concentration). Human Caco-2 intestinal epithelial cells were obtained from European Collection of Cell Cultures (ECACC, UK) and grown routinely in Dulbecco’s Minimum Essential Medium (DMEM), supplemented with 10% fetal bovine serum (FBS), 1% non-essential amino acid and 1% penicillin–streptomycin solution.

### Vancomycin aglycone hexapeptide (2)

The preparation of the title compound was based on literature procedures^29,30,33^. Vancomycin hydrochloride (**1**) (1.0 g, 0.673 mmol) was added to 30 mL of pyridine:water 1:1 mixture. Phenyl isothiocyanate (242 μL, 2.019 mmol, 3 equiv.) was added and the reaction mixture was stirred for 3 h at room temperature. Then, 200 mL of acetone was added, and the white precipitate was filtered off and washed with an additional 100 mL of acetone, 100 mL of diethyl ether, then dried. The crude material was dissolved in 10 mL of dry TFA and stirred for one hour at room temperature. Then, 1 mL of water was added and the reaction mixture was heated at 50 °C for 3 h. The mixture was concentrated to a smaller volume and the product was precipitated by the addition of diethyl ether (100 mL), filtered off and washed several times with diethyl ether. The crude product was dissolved in water (75 mL) and the pH was set to ~ 5–6 by adding 1 N sodium hydroxide solution. A yellowish green suspension formed which was filtered, washed with additional water, then after drying, with acetonitrile (50 mL) and ether (50 mL). TLC (Normal Phase Silica, MeCN:H_2_O = 87:13) indicated, that the cleaved carbohydrates were washed away, there was one glycopeptide derivative and an unknown yellow colored impurity. The yield of the crude product was 600 mg (70%) yellowish solid. MALDI-TOF m/z: 1038.261 [M+Na]^+^ (calcd. 1038.172 for C_46_H_39_Cl_2_N_7_O_16_Na); R_f_: 0.35 (Normal Phase Silica, MeCN:H_2_O = 87:13).

### *N*-decanoyl-D-Leu (6)

Decanoic acid **4** (2 g, 11.6 mmol) was dissolved in thionyl chloride (3 mL) and stirred while heating at 80 °C for two hours. After cooling to room temperature, the reaction mixture was co-evaporated with toluene in vacuo 3 times to remove the excess thionyl chloride. D-leucine **3** (787 mg, 6 mmol) was dissolved in a mixture of 1 M NaOH (6 mL) and THF (6 mL). The freshly prepared decanoyl chloride **5** (1.6 mL, about 1.3 equiv.) was dissolved in 3 mL of THF, and slowly added to the reaction mixture. After stirring for 5 h at room temperature, the solution was diluted with water (100 mL), acidified by cc. HCl solution (750 μL) and extracted with ethyl acetate (3 × 60 mL). The combined organic phase was dried on Na_2_SO_4_, filtered and evaporated to dryness at reduced pressure. The white residue was suspended in 50 mL of *i*-hexane and stirred for half an hour, after which it was filtered off and washed with an additional 50 mL of *i*-hexane, and dried in vacuum. Yield: 1.40 g (81%), white powder. MALDI-TOF m/z: 308.642 [M+Na]^+^ (calcd. 308.220 for C_16_H_31_NO_3_Na); ^1^H NMR (360 MHz, CDCl_3_) δ 11.22 (s, 1H), 6.32 (d, *J* = 8.1 Hz, 1H), 4.63 (td, *J* = 8.4, 4.5 Hz, 1H), 2.25 (t, *J* = 7.5 Hz, 2H), 1.77–1.66 (m, 2H), 1.66–1.53 (m, 3H), 1.33–1.24 (m, 10H), 0.95 (d, *J* = 4.6 Hz, 6H), 0.88 (t, *J* = 6.6 Hz, 3H). ^13^C NMR (91 MHz, CDCl_3_) δ 176.55, 174.44, 77.16, 50.95, 41.39, 36.57, 31.96, 29.56, 29.42, 29.36, 29.30, 25.78, 24.99, 22.94, 22.76, 21.99, 14.20. R_f_: 0.62 (Normal Phase Silica, MeCN:H_2_O = 85:15).

### *N*-decanoyl norvancomycin aglycone (7)

*N*-Decanoyl-D-Leu (**6**) (285 mg, 1 mmol) was dissolved in dichloromethane (3 mL) and a suspension of EDCI.HCl (211 mg, 1.1 mmol) in DCM (2 mL) was added dropwise to the solution. After stirring for 30 min at room temperature, *N*-hydroxysuccinimide (127 mg, 1.1 mmol) was added, and the stirring was continued for 18 h, after which TLC (*i*-hexane:ethyl acetate = 7:3) indicated almost total consumption of the starting material. The solvent was evaporated.

Crude vancomycin aglycone hexapeptide (**2**) (500 mg, ~ 0.5 mmol) was dissolved in DMF (4 mL). The above prepared crude NHS ester was dissolved in 1 mL of DMF and added to the reaction mixture. After 2 h of stirring at room temperature, TLC (Normal Phase Silica, toluene:methanol = 1:1) indicated the total consumption of **2**. Ethyl acetate (75 mL) and diethyl ether (75 mL) was added and the resulting precipitate was filtered off and washed with additional ether, then dried. The product (753 mg, yellow powder) was used in the next step in its crude form. MALDI-TOF m/z: 1305.361 [M+Na]^+^ (calcd. 1305.392 for C_62_H_68_Cl_2_N_8_O_18_Na); R_f_: 0.28 (Normal Phase Silica, toluene:MeOH = 1:1).

### *N*-Boc-ethylenediamine (9)

Ethylenediamine **8** (1.12 mL, 16.7 mmol, 6 equiv.) was added to DCM (5 mL). Boc_2_O (610 mg, 2.8 mmol) was dissolved in DCM (40 mL) in a dropping funnel and added to the reaction mixture in about 3 h at room temperature. Stirring was continued overnight, then the mixture was concentrated at reduced pressure. The crude material was dissolved in 2 M sodium carbonate solution (100 mL), and extracted with ethyl acetate (3 × 40 mL). The organic phase was dried on Na_2_SO_4_, filtered, concentrated then co-evaporated with toluene two times. Yield: 380 mg (85%) (colorless syrup). ^1^H NMR (400 MHz, CDCl_3_) δ 5.14 (s, 1H), 3.17 (q, *J* = 5.5 Hz, 2H), 2.79 (t, *J* = 5.9 Hz, 2H), 1.45 (s, 9H), 1.39 (s, 2H). ^13^C NMR (101 MHz, CDCl_3_) δ 156.30, 79.14, 43.43, 41.90, 28.45. R_f_: 0.27 (Normal Phase Silica, *i*-hexane:acetone = 6:4 + 1% v/v Et_3_N).

### *N*-decanoyl norvancomycin aglycone *N*-Boc-aminoethyl amide (10)

Compound **7** (500 mg, 0.39 mmol) was dissolved in 5 mL of DMF:DMSO 1:1 mixture, then Et_3_N (55 μL, 0.39 mmol) and **9** (125 mg, 0.78 mmol) were added, followed by PyBOP (203 mg, 0.39 mmol). After stirring for 3 h at room temperature, the starting material was consumed as indicated by TLC (Normal Phase Silica, toluene:methanol = 6:4). Diethyl ether (100 mL) was added, and the precipitate was filtered off and washed several times with ether. The resulting yellow material was dissolved in methanol, flash silica gel was added (Kieselgel 60) and the solvents were removed under reduced pressure. The product was purified by flash column chromatography using gradient elution (toluene:MeOH = 90:10, 85:15, 80:20, 75:25). Yield: 199 mg, yellow powder (25% from vancomycin·HCl) MALDI-TOF m/z: 1447.45 [M+Na]^+^ (calcd. 1447.50 for C_69_H_82_Cl_2_N_10_O_19_Na); R_f_: 0.63 (Normal Phase Silica, toluene:MeOH = 6:4).

### *N*-Boc-bromoethylamine (12)

Boc_2_O (2.18 g, 10 mmol) was dissolved in DCM (50 mL) and cooled to 0 °C. 2-bromoethylamine hydrobromide **11** (2 g, 10 mmol) was added, followed by the dropwise addition of Et_3_N (2.1 mL, 15 mmol). The reaction mixture was allowed to warm up to room temperature, and was stirred overnight. Then, it was diluted with DCM (400 mL) and washed with sat. aq. NH_4_Cl (2 × 50 mL), sat. aq. NaHCO_3_ (2 × 50 mL) and brine (2 × 50 mL). The combined organic layers were dried over anhydrous Na_2_SO_4_, filtered and concentrated under reduced pressure. The resulting syrup was chromatographed by flash column chromatography using gradient elution (*i*-hexane:acetone = 99:1, 97:3, 94:6) yielding 1.78 g (84%) of **12** as a white solid. ^1^H NMR (400 MHz, CDCl_3_) δ 5.02 (br s, 1H, N*H*), 3.57–3.49 (m, 2H, C*H*_*2*_), 3.45 (tr, *J* = 5.5 Hz, 2H, C*H*_*2*_), 1.45 (s, 9H, 3 × C*H*_*3*_). ^13^C NMR: δ 155.70 (*C*O), 79.85 (C_q_), 42.45 (NH-*C*H_2_), 32.83 (Br-*C*H_2_), 28.43 (3C, 3 × *C*H_3_). Rf: 0.59 (Normal Phase Silica, *i*-hexane:EtOAc = 8:2).

### Tetraalkyl *N*-decanoyl norvancomycin aglycone *N*-Boc-aminoethyl amide (13)

Compound **10** (200 mg, 0.14 mmol) was dissolved in DMF (2 mL). Cs_2_CO_3_ (684 mg, 2.1 mmol, 15 equiv.) was added and the reaction mixture was stirred for 30 min at room temperature followed by the addition of *N*-Boc-bromoethylamine (**12**) (1.255 g, 5.6 mmol, 40 equiv.) in DMF (1 mL). After stirring for 16 h, the reaction mixture was diluted with methanol (10 mL), silica gel was added, and the mixture was evaporated to dryness at reduced pressure. Purification started with flash chromatography using gradient elution (toluene:MeOH = 99:1, 95:5, 90:10, 85:15, 80:20). Further purification was carried out by dissolving the product in a DCM:MeOH 1:1 mixture, then applying it to a preparative TLC plate. (Normal Phase Silica, layer thickness: 1 mm, EtOAc:toluene:MeOH = 6:3:1 was used as eluent.) Yield: 88 mg (32%) white powder. MALDI-TOF m/z: 2019.78 [M+Na]^+^ (calcd. 2019.88 for C_97_H_134_Cl_2_N_14_O_27_Na);

### Tetraaminoethyl *N*-decanoyl norvancomycin aglycone aminoethyl amide trifluoroacetate salt (14)

Compound **13** was dissolved in dry TFA (1 mL) and stirred at room temperature for 2 h. Then, diethyl ether (50 mL) was added, the white precipitate was filtered off, and washed with additional 50 mL of ether, then dried. The solid was dissolved in H_2_O:MeCN = 9:1 + 0.2%v/v TFA, silanized silica was added and the slurry was applied to a silanized silica gel column in the same solvent mixture. A step gradient was used (H_2_O:MeCN = 85:15, 8:2, 7:3, 6:4 + 0.2%v/v TFA) for elution, clean fractions were collected, *n*-BuOH was added and the solvents were evaporated under reduced pressure (co-evaporated with toluene to remove TFA). The solid material was dissolved in water and lyophilized overnight to obtain **14** as a white foam. Yield: 64 mg (78%, HPLC purity 96%); MALDI-TOF m/z: 1519.60 (calcd. 1519.62 for C_72_H_94_Cl_2_N_14_O_17_Na); R_f_: 0.28 (Silanized Silica, H_2_O:MeCN = 6:4 + 0.5%v/v TFA).

### Cytotoxicity experiments

The human immortalized hCMEC/D3 cells (Merck KGaA, Darmstadt, Germany, Cat. # SCC066) were cultured in EndoGRO-MV Complete Culture Media (supplemented with the components of the kit and fibroblast growth factor 2 (FGF-2) at 1 ng/mL final concentration).

Human Caco-2 intestinal epithelial cells were obtained from European Collection of Cell Cultures (ECACC, UK) and grown routinely in Dulbecco’s Minimum Essential Medium (DMEM), supplemented with 10% fetal bovine serum (FBS), 1% non-essential amino acid and 1% penicillin–streptomycin solution.

In cell viability experiments, 1 × 10^4^ hCMEC/D3 or Caco-2 cells/well were seeded on 96-weel plates. After 24 h of incubation, cells were treated with test solutions in different concentrations. The stock solution of the test molecule was dissolved in DMSO and further diluted with cell culture medium to prepare test solutions. The final concentration of DMSO in the test solutions did not exceed 0.5 V/V%. The control group received culture medium. Cells were incubated with the test solutions for 72 h at 37 °C in an atmosphere of 5% CO2, and test solutions were replaced with 0.05 mg/ml 3-(4,5-dimethylthiazol-2-yl)-2,5-diphenyltetrazolium bromide (MTT) solutions prepared in PBS. Cells were incubated with MTT solutions for 4 h at 37 °C and the formed dark blue formazan crystals were dissolved in acidic isopropanol (isopropanol: 1.0 N hydrochloric acid = 25:1). The absorbance was measured at 570 nm against a 690 nm reference wavelength with a Thermo Fisher Multiskan Go microplate reader (Thermo Fisher, Waltham, MA, USA). Cell viability was expressed as the percentage of the untreated control and IC50 values were calculated by GraphPad Prism 7 software (GraphPad Software Inc., San Diego, CA, USA).

Cells were also treated with the combinations of derivative **14** and teicoplanin or vancomycin between 2 and 64 µg/mL in checkerboard configuration by the above detailed MTT test.

## Supplementary Information

Supplementary Information.

## Data Availability

The datasets used and/or analysed during the current study available from the corresponding author (A.B.) on reasonable request.
