# Supplementary material for: Synthesis of an amphiphilic vancomycin aglycone derivative inspired by polymyxins: overcoming glycopeptide resistance in Gram-positive and Gram-negative bacteria in synergy with teicoplanin in vitro
Source: Sci Rep. 2022 Dec 3;12:20921. doi: 10.1038/s41598-022-24807-0 (PMC9719540; doi:10.1038/s41598-022-24807-0)
Supplement: Supplementary file 1 — Supplementary Information. [file 41598_2022_24807_MOESM1_ESM.docx]

**Supplementary information**

**Synthesis of an Amphiphilic Vancomycin Aglycone Derivative Inspired by Polymyxins - Overcoming Glycopeptide Resistance in Gram-negative Bacteria in Synergy with Teicoplanin**

Zsolt Szűcs,^1^ Ilona Bereczki,^1^ Ferenc Fenyvesi,^2^ Pál Herczegh,^1^ Eszter Ostorházi,^3^ Anikó Borbás^1^

^1^Department of Pharmaceutical Chemistry, University of Debrecen, Egyetem tér 1, H-4032 Debrecen, Hungary

^2^Department of Pharmaceutical Technology, Faculty of Pharmacy, University of Debrecen, Nagyerdei Körút 98, Debrecen, H-4032, Hungary

^3^Department of Medical Microbiology, Semmelweis University, Nagyvárad tér 4, H-1089 Budapest, Hungary

Table of contents

[Combined antibacterial susceptibility tests	S2](#_Toc118722824)

[Isobolograms for combinations of compound **14** with teicoplanin and vancomycin	S10](#_Toc118722825)

[NMR spectra of compound **6**	S11](#_Toc118722826)

[NMR spectra of compound **9**	S12](#_Toc118722827)

[NMR spectra of compound **12**	S13](#_Toc118722828)

[NMR assignment of compound **14**	S14](#_Toc118722829)

[HPLC-MS of Compound **14**	S31](#_Toc118722830)

# Combined antibacterial susceptibility tests

Table S1. Combined susceptibility testing of *K. pneumoniae* in a Vancomycin/Compound **14** Checkboard with different concentrations in mg/L

|  | C14 128 | C14 64 | C14 32 | C14 16 | C14 8 | C14 4 | C14 2 | C14  1 |  |  | negative control | positive control |
| --- | --- | --- | --- | --- | --- | --- | --- | --- | --- | --- | --- | --- |
| Vancomycin 128 |  |  |  |  |  |  |  |  |  |  |  |  |
| Vancomycin 64 |  |  |  |  |  |  |  |  |  |  |  |  |
| Vancomycin 32 |  |  |  |  |  |  |  |  |  |  |  |  |
| Vancomycin 16 |  |  |  |  |  |  |  |  |  |  |  |  |
| Vancomycin  8 |  |  |  |  |  |  |  |  |  |  |  |  |
| Vancomycin 4 |  |  |  |  |  |  |  |  |  |  |  |  |
| Vancomycin 2 |  |  |  |  |  |  |  |  |  |  |  |  |
| Vancomycin 1 |  |  |  |  |  |  |  |  |  |  |  |  |

Figure S1. Combined susceptibility testing of *K. pneumoniae* in a Vancomycin/Compound **14** Checkboard with different concentrations in mg/L

Table S2. Combined susceptibility testing of *K. pneumoniae* in a Teicoplanin/Compound 14 Checkboard with different concentrations in mg/L

|  | C14 128 | C14 64 | C14 32 | C14 16 | C14 8 | C14 4 | C14 2 | C14  1 |  |  | negative control | positive control |
| --- | --- | --- | --- | --- | --- | --- | --- | --- | --- | --- | --- | --- |
| Teicoplanin 128 |  |  |  |  |  |  |  |  |  |  |  |  |
| Teicoplanin 64 |  |  |  |  |  |  |  |  |  |  |  |  |
| Teicoplanin 32 |  |  |  |  |  |  |  |  |  |  |  |  |
| Teicoplanin 16 |  |  |  |  |  |  |  |  |  |  |  |  |
| Teicoplanin  8 |  |  |  |  |  |  |  |  |  |  |  |  |
| Teicoplanin 4 |  |  |  |  |  |  |  |  |  |  |  |  |
| Teicoplanin 2 |  |  |  |  |  |  |  |  |  |  |  |  |
| Teicoplanin 1 |  |  |  |  |  |  |  |  |  |  |  |  |

Figure S2. Combined susceptibility testing of *K. pneumoniae* in a Teicoplanin/Compound **14** Checkboard with different concentrations in mg/L

Table S3. Combined susceptibility testing of *P. aeruginosa* in a Vancomycin/Compound **14** Checkboard with different concentrations in mg/L

|  | C14 128 | C14 64 | C14 32 | C14 16 | C14 8 | C14 4 | C14 2 | C14  1 |  |  | negative control | positive control |
| --- | --- | --- | --- | --- | --- | --- | --- | --- | --- | --- | --- | --- |
| Vancomycin 128 |  |  |  |  |  |  |  |  |  |  |  |  |
| Vancomycin 64 |  |  |  |  |  |  |  |  |  |  |  |  |
| Vancomycin 32 |  |  |  |  |  |  |  |  |  |  |  |  |
| Vancomycin 16 |  |  |  |  |  |  |  |  |  |  |  |  |
| Vancomycin  8 |  |  |  |  |  |  |  |  |  |  |  |  |
| Vancomycin 4 |  |  |  |  |  |  |  |  |  |  |  |  |
| Vancomycin 2 |  |  |  |  |  |  |  |  |  |  |  |  |
| Vancomycin 1 |  |  |  |  |  |  |  |  |  |  |  |  |

Figure S3. Combined susceptibility testing of *P. aeruginosa* in a Vancomycin/Compound **14** Checkboard with different concentrations in mg/L

Table S4. Combined susceptibility testing of *P. aeruginosa* in a Teicoplanin/Compound **14** Checkboard with different concentrations in mg/L

|  | C14 128 | C14 64 | C14 32 | C14 16 | C14 8 | C14 4 | C14 2 | C14  1 |  |  | negative control | positive control |
| --- | --- | --- | --- | --- | --- | --- | --- | --- | --- | --- | --- | --- |
| Teicoplanin 128 |  |  |  |  |  |  |  |  |  |  |  |  |
| Teicoplanin 64 |  |  |  |  |  |  |  |  |  |  |  |  |
| Teicoplanin 32 |  |  |  |  |  |  |  |  |  |  |  |  |
| Teicoplanin 16 |  |  |  |  |  |  |  |  |  |  |  |  |
| Teicoplanin  8 |  |  |  |  |  |  |  |  |  |  |  |  |
| Teicoplanin 4 |  |  |  |  |  |  |  |  |  |  |  |  |
| Teicoplanin 2 |  |  |  |  |  |  |  |  |  |  |  |  |
| Teicoplanin 1 |  |  |  |  |  |  |  |  |  |  |  |  |

Figure S4. Combined susceptibility testing of *P. aeruginosa* in a Teicoplanin/Compound **14** Checkboard with different concentrations in mg/L

Table S5. Combined susceptibility testing of *E.coli* in a Vancomycin/Compound **14** Checkboard with different concentrations in mg/L

|  | C14 128 | C14 64 | C14 32 | C14 16 | C14 8 | C14 4 | C14 2 | C14  1 |  |  | negative control | positive control |
| --- | --- | --- | --- | --- | --- | --- | --- | --- | --- | --- | --- | --- |
| Vancomycin 128 |  |  |  |  |  |  |  |  |  |  |  |  |
| Vancomycin 64 |  |  |  |  |  |  |  |  |  |  |  |  |
| Vancomycin 32 |  |  |  |  |  |  |  |  |  |  |  |  |
| Vancomycin 16 |  |  |  |  |  |  |  |  |  |  |  |  |
| Vancomycin  8 |  |  |  |  |  |  |  |  |  |  |  |  |
| Vancomycin 4 |  |  |  |  |  |  |  |  |  |  |  |  |
| Vancomycin 2 |  |  |  |  |  |  |  |  |  |  |  |  |
| Vancomycin 1 |  |  |  |  |  |  |  |  |  |  |  |  |

Figure S5. Combined susceptibility testing of *E.coli* in a Vancomycin/Compound **14** Checkboard with different concentrations in mg/L

Table S6. Combined susceptibility testing of *E.coli* in a Teicoplanin/Compound **14** Checkboard with different concentrations in mg/L

|  | C14 128 | C14 64 | C14 32 | C14 16 | C14 8 | C14 4 | C14 2 | C14  1 |  |  | negative control | positive control |
| --- | --- | --- | --- | --- | --- | --- | --- | --- | --- | --- | --- | --- |
| Teicoplanin 128 |  |  |  |  |  |  |  |  |  |  |  |  |
| Teicoplanin 64 |  |  |  |  |  |  |  |  |  |  |  |  |
| Teicoplanin 32 |  |  |  |  |  |  |  |  |  |  |  |  |
| Teicoplanin 16 |  |  |  |  |  |  |  |  |  |  |  |  |
| Teicoplanin  8 |  |  |  |  |  |  |  |  |  |  |  |  |
| Teicoplanin 4 |  |  |  |  |  |  |  |  |  |  |  |  |
| Teicoplanin 2 |  |  |  |  |  |  |  |  |  |  |  |  |
| Teicoplanin 1 |  |  |  |  |  |  |  |  |  |  |  |  |

Figure S6. Combined susceptibility testing of *E.coli* in a Teicoplanin/Compound **14** Checkboard with different concentrations in mg/L

Table S7. Combined susceptibility testing of *A. baumannii* in a Vancomycin/Compound **14** Checkboard with different concentrations in mg/L

|  | C14 128 | C14 64 | C14 32 | C14 16 | C14 8 | C14 4 | C14 2 | C14  1 |  |  | negative control | positive control |
| --- | --- | --- | --- | --- | --- | --- | --- | --- | --- | --- | --- | --- |
| Vancomycin 128 |  |  |  |  |  |  |  |  |  |  |  |  |
| Vancomycin 64 |  |  |  |  |  |  |  |  |  |  |  |  |
| Vancomycin 32 |  |  |  |  |  |  |  |  |  |  |  |  |
| Vancomycin 16 |  |  |  |  |  |  |  |  |  |  |  |  |
| Vancomycin  8 |  |  |  |  |  |  |  |  |  |  |  |  |
| Vancomycin 4 |  |  |  |  |  |  |  |  |  |  |  |  |
| Vancomycin 2 |  |  |  |  |  |  |  |  |  |  |  |  |
| Vancomycin 1 |  |  |  |  |  |  |  |  |  |  |  |  |

Figure S7. Combined susceptibility testing of *A. baumannii* in a Vancomycin/Compound **14** Checkboard with different concentrations in mg/L

Table S8. Combined susceptibility testing of *A. baumannii* in a Teicoplanin/Compound **14** Checkboard with different concentrations in mg/L

|  | C14 128 | C14 64 | C14 32 | C14 16 | C14 8 | C14 4 | C14 2 | C14  1 |  |  | negative control | positive control |
| --- | --- | --- | --- | --- | --- | --- | --- | --- | --- | --- | --- | --- |
| Teicoplanin 128 |  |  |  |  |  |  |  |  |  |  |  |  |
| Teicoplanin 64 |  |  |  |  |  |  |  |  |  |  |  |  |
| Teicoplanin 32 |  |  |  |  |  |  |  |  |  |  |  |  |
| Teicoplanin 16 |  |  |  |  |  |  |  |  |  |  |  |  |
| Teicoplanin  8 |  |  |  |  |  |  |  |  |  |  |  |  |
| Teicoplanin 4 |  |  |  |  |  |  |  |  |  |  |  |  |
| Teicoplanin 2 |  |  |  |  |  |  |  |  |  |  |  |  |
| Teicoplanin 1 |  |  |  |  |  |  |  |  |  |  |  |  |

Figure S8. Combined susceptibility testing of *A. baumannii* in a Teicoplanin/Compound **14** Checkboard with different concentrations in mg/L

# Isobolograms for combinations of compound 14 with teicoplanin and vancomycin

Teicoplanin + compound 14

Teicoplanin mg/L

| compound 14 mg/L | K. pneumoniae | A. baumannii | E. coli | P. aeruginosa |
| --- | --- | --- | --- | --- |
| 512 | 0 |  |  |  |
| 512 | 1 |  |  |  |
| 512 | 2 |  |  |  |
| 512 | 4 |  |  |  |
| 512 | 8 |  |  |  |
| 512 | 16 |  |  |  |
| 512 | 32 |  | 0 | 0 |
| 512 | 64 |  | 1 | 1 |
| 512 | 128 |  | 2 | 2 |
| 512 | 256 |  | 4 | 4 |
| 512 | 512 |  | 8 | 8 |
| 256 | 512 | 0 | 16 | 16 |
| 256 | 512 | 1 | 16 | 16 |
| 128 | 512 | 2 | 32 | 32 |
| 64 | 512 | 2 | 32 | 64 |
| 32 | 512 | 32 | 32 | 128 |
| 16 | 512 | 256 | 64 | 256 |
| 8 | 512 | 256 | 64 | 256 |
| 4 | 512 | 256 | 128 | 256 |
| 2 | 512 | 256 | 128 | 256 |
| 1 | 512 | 256 | 256 | 256 |
| 0 | 512 | 256 | 256 | 256 |

Figure S9. Isobologram for compound **14** and teicoplanin mixtures tested against *Klebsiella pneumoniae* ST258 clone K 160/09, *Acinetobacter* *baumannii* ATCC BAA1605, *Escherichia* *coli* ATCC 25218 and *Pseudomonas* *aeruginosa* ATCC 27853

Vancomycin + compound 14

Vancomycin mg/L

| compound 14 mg/L | K. pneumoniae | A. baumannii | E. coli | P. aeruginosa |
| --- | --- | --- | --- | --- |
| 512 | 0 |  |  |  |
| 512 | 1 |  |  |  |
| 512 | 2 |  |  |  |
| 512 | 4 |  |  |  |
| 512 | 8 |  |  |  |
| 512 | 16 |  |  | 0 |
| 512 | 32 |  |  | 1 |
| 512 | 64 |  |  | 2 |
| 512 | 128 |  |  | 4 |
| 512 | 256 |  |  | 8 |
| 512 | 512 |  | 0 | 16 |
| 256 | 512 | 0 | 32 | 32 |
| 256 | 512 | 1 | 32 | 32 |
| 256 | 512 | 2 | 32 | 32 |
| 256 | 512 | 4 | 32 | 32 |
| 256 | 512 | 8 | 32 | 32 |
| 256 | 512 | 16 | 32 | 32 |
| 128 | 512 | 32 | 64 | 64 |
| 64 | 512 | 64 | 64 | 64 |
| 32 | 512 | 256 | 64 | 128 |
| 16 | 512 | 256 | 64 | 128 |
| 8 | 512 | 256 | 128 | 256 |
| 4 | 512 | 256 | 128 | 256 |
| 2 | 512 | 256 | 128 | 256 |
| 1 | 512 | 256 | 256 | 256 |
| 0 | 512 | 256 | 256 | 256 |

Figure S10. Isobologram for compound **14** and vancomycin mixtures tested against *Klebsiella pneumoniae* ST258 clone K 160/09, *Acinetobacter* *baumannii* ATCC BAA1605, *Escherichia* *coli* ATCC 25218 and *Pseudomonas* *aeruginosa* ATCC 27853

# NMR spectra of compound 6

Figure S11. ^1^H NMR spectrum of compound **6**

Figure S12. ^13^C NMR spectrum of compound **6**

# NMR spectra of compound 9

Figure S13. ^1^H NMR spectrum of compound **9**

Figure S14. ^13^C NMR spectrum of compound **9**

# NMR spectra of compound 12

Figure S15. ^1^H NMR spectrum of compound **12**

Figure S16. ^1^H NMR spectrum of compound **12**

# NMR assignment of compound 14

Figure S17. Atom numbering for compound **14**

Figure S18. ^1^H-^13^C HSQC NMR spectrum of compound **14** (1/4)

Figure S19. ^1^H-^13^C HSQC NMR spectrum of compound **14** (2/4)

Figure S20. ^1^H-^13^C HSQC NMR spectrum of compound **14** (3/4)

Figure S21. ^1^H-^13^C HSQC NMR spectrum of compound **14** (4/4)

Figure S22. ^1^H-^13^C HMBC NMR spectrum of compound **14** (1/2)

Figure S23. ^1^H-^13^C HMBC NMR spectrum of compound **14** (2/2)

Figure S24. ^1^H-^1^H COSY NMR spectrum of compound **14** (1/3)

Figure S25. ^1^H-^1^H COSY NMR spectrum of compound **14** (2/3)

Figure S26. ^1^H-^1^H COSY NMR spectrum of compound **14** (3/3)

Figure S27. ^1^H-^1^H NOESY NMR spectrum of compound **14** (1/4)

Figure S28. ^1^H-^1^H NOESY NMR spectrum of compound **14** (2/4)

Figure S29. NOEs for compound **14** (3a)

Figure S30. ^1^H-^1^H NOESY NMR spectrum of compound **14** (3/4)

Figure S31. NOEs for compound **14** (F1)

Figure S32. ^1^H-^1^H NOESY NMR spectrum of compound **14** (4/4)

Figure S33. NOEs for compound **14** (side chains G, H, I)

# HPLC-MS of Compound 14

Figure S34. Total ion chromatogram

Table S9. HPLC-MS peak list

| Peak | RT [min] | Area | Mass | Area Frac. % |
| --- | --- | --- | --- | --- |
| 1 | 1.50 | 51851 |  | 0.5 |
| 2 | 1.76 | 6104156 | 499.884 | 96.0 |
| 3 | 2.30 | 3306318 | 499.884 |  |
| 4 | 3.34 | 346708 |  | 3.5 |

Figure S35. Mass spectrum/isotopic distribution of compound **14**
